# “It’s Brought Me a Lot Closer to Who I Am”: A Mixed Methods Study of Posttraumatic Growth and Positive Change Following a First Episode of Psychosis

**DOI:** 10.3389/fpsyt.2019.00480

**Published:** 2019-07-15

**Authors:** Gerald Jordan, Ashok Malla, Srividya N. Iyer

**Affiliations:** ^1^Prevention and Early Intervention Program for Psychosis, Douglas Mental Health University Institute, Verdun, QC, Canada; ^2^Department of Psychiatry, McGill University, Montreal, QC, Canada; ^3^Program for Recovery and Community Health, Yale University, New Haven, CT, United States

**Keywords:** posttraumatic growth, positive change, first-episode psychosis, mixed methods, youth, early intervention

## Abstract

**Background:** A first episode of psychosis is often a traumatic experience that may also lead to positive change, a phenomenon that has received little attention. This knowledge gap may impede service providers’ capacity to foster positive change among service users.

**Objective:** To investigate aspects of positive change among persons receiving early intervention services for psychosis.

**Design:** The study objective was addressed using a mixed methods convergent design, which entailed simultaneously employing qualitative and quantitative methods.

**Setting:** This study was conducted at a specialized early intervention service for psychosis based in Montreal, Quebec, Canada.

**Participants:** Participants included service users receiving services at an early intervention service for psychosis. Participants had to be fluent in English or French, be clinically stable enough to take part in the study, and have received at least 6 months of treatment. Participants were conveniently sampled in the quantitative component and purposefully sampled in the qualitative component. The quantitative component was carried out using a cross-sectional survey design. Ninety-four participants completed the Posttraumatic Growth Inventory, a widely used measure of positive change. Data on the extent and domains of posttraumatic growth were summarized using descriptive statistics. The qualitative component was carried out using a qualitative descriptive approach. Semistructured interviews were conducted with 12 participants. Data were analyzed using thematic analysis. Findings from both components were integrated using a weaving method in the discussion section.

**Results:** Quantitative results indicated that most participants reported a moderate amounts of posttraumatic growth. A greater appreciation of life was the most commonly endorsed domain, whereas spiritual growth was the least commonly endorsed domain. The qualitative results revealed that in addition to suffering, participants experienced positive changes, such as improved health and personality, and a stronger sense of self; stronger, more balanced religiosity and spirituality; improved relationships with others; and improved lifestyles, goals, and expectations for the future.

**Conclusions:** Positive change may be a common phenomenon in the aftermath of first episode psychosis. The study findings may provide hope to those who have experienced a first episode of psychosis and can inform efforts by early intervention services to provide recovery-oriented, growth-focused care.

## Introduction

The onset of psychosis is often traumatic and marked by significant suffering (1, 2). Most research on first-episode psychosis (FEP) has focused on its negative aftermath. However, similar to what has been observed following major life-threatening events and physical illnesses (3), persons may also experience positive changes following the onset of psychosis (4, 5).

The most commonly applied model of positive change is that of posttraumatic growth, which has been defined as a fundamental qualitative shift in functioning following a highly negative experience (6–8). Aspects of posttraumatic growth include a greater appreciation for life, improved ways of relating to others, greater personal strength, greater spirituality, and new life possibilities.

Posttraumatic growth has been reported among persons who have experienced multiple episodes of psychosis (9, 10). In our scoping and systematic reviews on positive change following FEP, we identified only three studies that had specifically focused on positive change following FEP , and 37 studies which described positive change as part of other processes (e.g., recovery, helpseeking, etc.) These changes occurred at the individual (e.g., greater insight and clarity), interpersonal (e.g., improved relationships), and spiritual (e.g., greater religiosity) levels (11–13). Two studies included in these reviews used qualitative methods to investigate posttraumatic growth following FEP (11, 12). The only quantitative study investigating posttraumatic growth following FEP (13) had limited generalizability because it relied on a small number of participants.

Furthermore, the three studies explicitly examining posttraumatic growth following FEP were conducted outside the context of early intervention services for psychosis, which are internationally recognized as a desirable avenue for service delivery in early psychosis. Early intervention services are predicated on a philosophy of hope and therapeutic optimism and, therefore, may be likely to contribute to perceptions of positive change. Addressing this knowledge gap may aid early intervention services in developing strengths-based and resilience-enhancing interventions (14, 15). To address this gap, this study investigated positive change among youth receiving early intervention services for FEP using mixed methods.

## Methods and Measures

The study objective was addressed using a mixed methods convergent design (16), whereby qualitative and quantitative components were conducted separately, simultaneously, and with equal priority (17). We used mixed methods for its potential to yield a more complete understanding of positive change following FEP and to capitalize on the strengths of both qualitative and quantitative approaches. The study was approved by McGill University’s Research Ethics Board.

### Paradigmatic Stance

Science is guided by philosophical paradigms. Typically, quantitative research is guided by a post-positivistic paradigm that is based on the existence of an objective measurable reality that is shaped to a lesser or greater degree by subjective experiences. Qualitative research is often guided by constructivist paradigms that acknowledge the existence of multiple truths that are shaped by varying contexts (e.g., social, cultural, etc.).

The quantitative component of this study was guided by a post-positivistic paradigm while a constructivist paradigm guided the qualitative component (16, 17). Tensions exist in combining paradigms such as these within a single mixed methods study. As a solution, dialectical pluralism was the overarching paradigm guiding this research, which is a metaparadigm that helps researchers feel comfortable living with the tensions inherent in combining multiple paradigms within a single study.

### Study Setting

Participants were service users recruited from the Prevention and Early Intervention Program for Psychoses, which is a catchment area-based early intervention service treating all referred cases of FEP in Montreal, Canada. Persons are accepted to the service if they are between the ages of 14 and 35, have an IQ of at least 70, are experiencing a nonaffective or affective psychotic disorder that is not caused by an organic brain disorder (e.g., epilepsy) or induced entirely by substance use, and have not taken antipsychotic medication for more than 30 days (15).

### Eligibility Criteria

To participate in the quantitative component of the study, service users needed to have completed at least 6 months but no more than 5 years of treatment, be at least 18 years of age, be fluent in either English or French, and be clinically stable enough to participate in the study (i.e., not be experiencing a relapse based on their treatment team’s report). To participate in the qualitative component, service users needed to have met the above criteria and had to have been identified by their treatment team (e.g., case manager) as potentially having experienced positive change following their FEP.

### Data Collection

#### Quantitative Methods

A cross-sectional survey design, whereby participants were administered questionnaires at a single time point during their follow-up, was used to carry out the quantitative component of the study. The total number of participants was based on a power calculation (16).

#### Quantitative Assessments

Questionnaires were completed online or using pen and paper, in English or French, depending on the participant’s preference.

Participants completed a demographic questionnaire and the Posttraumatic Growth Inventory, which is a well-validated, widely used measure of positive change following adversity (18). The Posttraumatic Growth Inventory has 21 items rated on a six-point Likert-type scale. It measures positive change in five domains, including relating to others (e.g., I have more compassion for others); personal strength (e.g., I have a greater feeling of self-reliance); appreciation for life (e.g., I can better appreciate each day); spiritual change (e.g., I have a stronger religious faith); and new possibilities (e.g., I developed new interests). The scale was adapted to measure positive change following participants’ “mental health problem.” Participants were asked to rate how they changed as a result of the mental health problem for which they received early intervention services (i.e., psychosis). French-speaking participants completed a French version of the demographic questionnaire and the Posttraumatic Growth Inventory. Both questionnaires had been translated by our group based on World Health Organization recommendations (19).

Trained research staff completed several clinical measures as part of the study site’s ongoing data collection protocol. The Circumstances of Onset and Relapse Schedule, a validated semistructured interview conducted with patients (and families, where possible), was used to assess the age of onset of psychosis (20). The Scale for the Assessment of Positive Symptoms (21) and the Scale for the Assessment of Negative Symptoms (22) were used to assess positive and negative symptoms, respectively. Diagnoses were made using the Structured Clinical Interview for DSM-IV within the first 3 months of follow-up by trained staff. SCID-IV diagnoses were based on consensus between the research team, an experienced psychiatrist, and the interviewer.

#### Qualitative Methods

A qualitative descriptive approach was used to provide a rich account of a phenomenon of interest close to the words of participants. Such descriptions are to some degree shaped by interpretation, theory, or the naturalistic background (e.g., their clinical experience, etc.) of the investigators (23, 24).

Participants were purposefully sampled according to whether their treatment team felt that they had experienced positive change following FEP. Recruitment ended at thematic saturation (i.e., when no new themes related to how participants experienced positive change emerged from the interviews).

Data were collected through semistructured interviews lasting approximately 1 hour in English using an interview guide developed through consultation with relevant stakeholders (i.e., service users, family members, clinicians, and researchers). The interviews sought to determine how participants came to receive early intervention services, how they changed because of those challenges, and what they felt facilitated such changes. However, interviews remained open to what participants felt was important to discuss regarding the topics. Detailed notes were written following each interview. Interviews were audiotaped, transcribed verbatim, and checked for accuracy by two researchers. The first author engaged in extensive reflexive practice (25, 26) to examine the impact of his “multiple brought selves” on the research process (27, 28) (e.g., how his role as a staff member at the early intervention service may have shaped the interviews, etc.).

## Results

### Quantitative Data Analysis

Preliminary analyses were conducted to detect differences in posttraumatic growth scores according to the questionnaire’s language of administration. Skewed variables were corrected with appropriate transformations.

Descriptive statistics were calculated for the Posttraumatic Growth Inventory. Item means (i.e., the average response across the entire Posttraumatic Growth Inventory and its subscales) were also computed.

Consistent with prior reporting (29), responses to items of the Posttraumatic Growth Inventory were grouped according to whether they reflected “no to a small amount” of posttraumatic growth (i.e., a score of ≤3) or a “moderate to great amount” of posttraumatic growth (i.e., a score of ≥3).

Correlations between positive (i.e., hallucinations, delusions, bizarre behavior, and thought disorder) and negative symptom (i.e., affective flattening, alogia, apathy, avolition, and anhedonia) global scores with posttraumatic growth were computed.

### Qualitative Data Analysis

A thematic analysis was conducted to develop themes depicting the ways participants changed following FEP (30). Entire transcripts were subject to line-by-line open coding. Coding was directed, first and foremost, by what participants said themselves. In addition, coding was informed by theoretical constructions of positive change (6–8, 31–34), two systematic reviews on this topic (4, 5), and the author’s clinical and personal experiences with people with FEP. Codes were combined into focused codes, subthemes, and themes, which were reviewed and compared to codes and text to check for internal consistency. Analytical memos, thoughts, reflections, and reflexive notes were read and reflected upon to help shape the analyses. Transcripts were read and reread multiple times to develop a narrative from the themes.

### Mixed Methods Data Analysis

As per expert recommendations (35), convergence and divergence between the qualitative and quantitative findings were integrated as a narrative in the discussion section using a “weaving” approach (36).

### Participants

Between May 2015 and November 2017, 147 service users receiving services at the study site were approached to participate in the quantitative component: 36 declined and 111 participated. Data from 16 participants were excluded for various reasons (i.e., they did not return questionnaires, did not complete the questionnaires as per the instructions, or they completed pilot versions of the questionnaires), and one participant withdrew consent, yielding a final sample of 94.

During the same study period, 14 service users were approached to participate in the qualitative component. One service user declined, 13 completed interviews, and one withdrew consent from the study, yielding a final sample size of 12. All participants who completed the qualitative component also completed the quantitative component.

### Quantitative Results

Demographic and clinical characteristics of participants are presented in **Table 1**.

**Table 1:** Baseline demographic and clinical characteristics of participants who completed interviews and questionnaires and participants who only completed questionnaires.

Variable	Participants who completed questionnaires and interviews (*n* = 12) M/SD; *f*/%	Participants who completed questionnaires (*n* = 94) M/SD; *f*/%
Age at assessment	24.27 (2.76)	25.52 (5.11)
Age of psychosis onset	22.17 (6.14)	23.27 (5.45)
Gender (female)	5 (41.6%)	40 (44.0%)
Education (at least high school)	11 (91.7%)	64 (76.2%)
Relationship status (in a relationship)	3 (25%)	9 (10.1%)
Visible minority (yes)	7 (58.3%)	37 (45.7)
Born outside Quebec (yes)	7 (58.3%)	31 (35.2%)
*Socioeconomic status (middle to upper class)	9 (75.0%)	26 (38.8%)
Income derived from paid employment (yes)	6 (50.0%)	19 (24.1%)
Living with friends, family, or independently	11 (91.67%)	81 (96.4%)
SCID-IV baseline schizophrenia-spectrum diagnosis (yes)	6 (50.0%)	55 (63.2%)
SCID-IV baseline diagnosis of substance abuse/dependence (yes)	4 (33.3%)	27 (34.6%)
Baseline positive symptoms (SAPS)	14.00 (2.75)	11.84 (3.26)
Baseline negative symptoms (SANS)	9.45 (3.55)	10.39 (3.60)

Socioeconomic status was measured using the Hollingshead Four Factor Index of Socioeconomic Status.

No significant differences in posttraumatic growth were detected because of questionnaires being completed in English (*n* = 62) versus French (*n* = 32).

No significant correlations were observed between posttraumatic growth and hallucinations (*r* = −0.08, *P* = 0.50), delusions (*r* = 0.11, *P* = 0.35), thought disorder (*r* = 0.15, *P* = 0.31), bizarre behavior (*r* = 0.09, *P* = 0.43), affective flattening (*r* = −0.10, *P* = 0.38), alogia (*r* = 0.10, *P* = 0.38), avolition (*r* = 0.14, *P* = 0.24), and anhedonia (*r* = −0.06, *P* = 0.62).

The average score on the Posttraumatic Growth Inventory was 59.40 (SD = 26.81). Scores ranged between 0 and 105, and the distribution of responses was normal (skewness = −0.13) (**Table 2**). Participants indicated experiencing posttraumatic growth following FEP on all domains of the Posttraumatic Growth Inventory, which included new ways of relating to others (M = 19.66, SD = 10.00), new possibilities (M = 13.96, SD = 6.84), greater personal strength (M = 11.59, SD = 5.48), spiritual changes (M = 4.75, SD = 3.70), and new appreciation for life (M = 9.40, SD = 4.02). Examining item means of the Posttraumatic Growth Inventory revealed that participants reported moderate amounts of posttraumatic growth across all items (M = 2.92, variance = 0.06).

**Table 2:** Descriptive statistics pertaining to the Posttraumatic Growth Inventory.

Posttraumatic growth domain	Number of items	Item mean	Scale mean	Standard deviation	Median	Range*	Skewness
Relating to Others	7	2.85	19.66	10.00	21.00	35.00	−0.17
New Possibilities	5	2.82	13.96	6.84	15.00	25.00	−0.17
Personal Strength	4	2.95	11.59	5.48	12.00	20.0	−0.30
Spiritual Change	2	2.70	4.78	3.70	5.00	10.00	0.03
Appreciation of Life	3	3.16	9.40	4.02	9.50	15.00	−0.37
Total	21	2.92	59.40	26.81	58.50	105.00	−0.13

*Represents the actual range.

**Figure 1:**
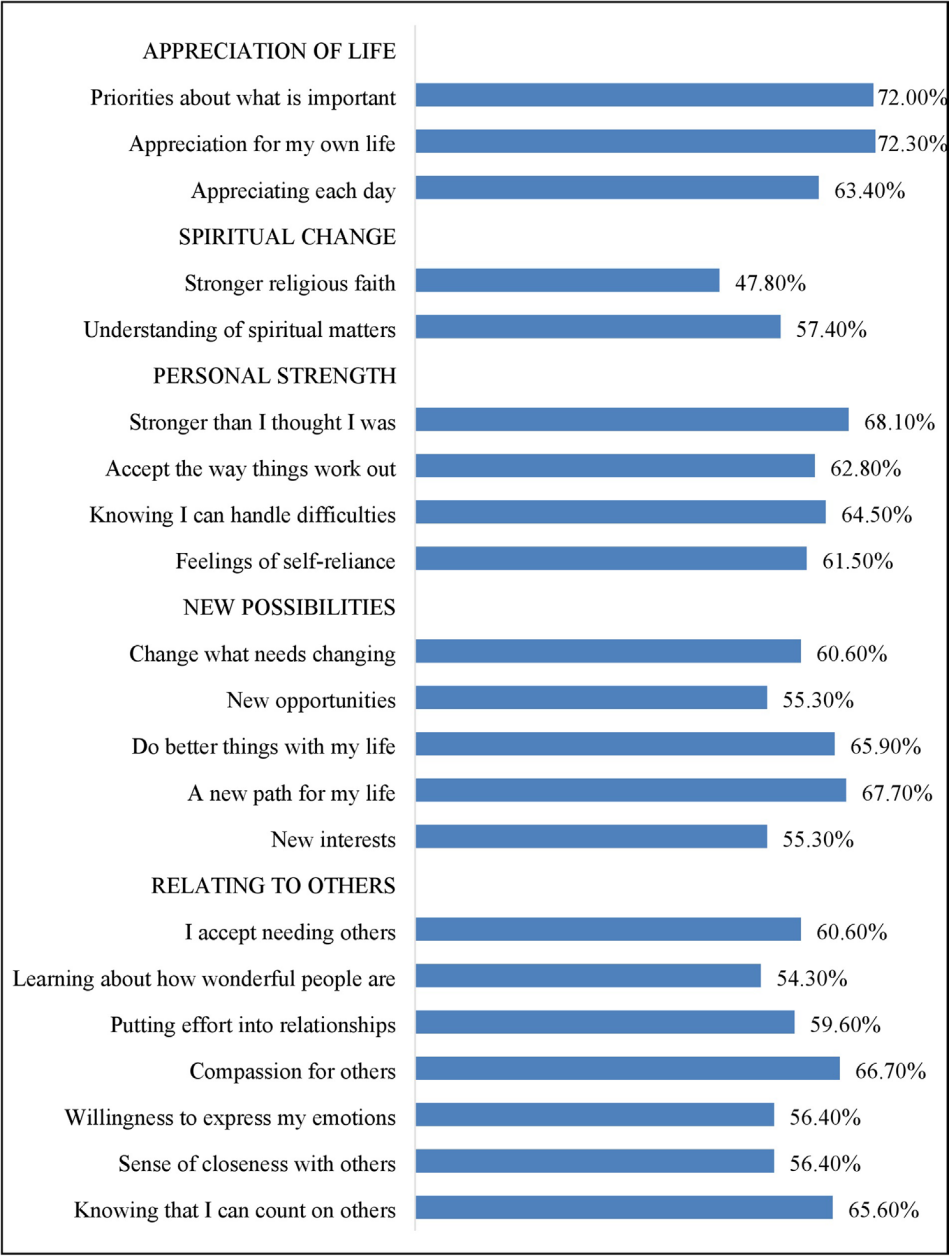
Proportion of participants endorsing moderate to very great change on each item of the Posttraumatic Growth Inventory (organized under the scale’s five domains).

Finally, between 50 and 75% of participants endorsed “moderate to great” posttraumatic growth on each item of the Posttraumatic Growth Inventory. The item assessing stronger religious faith received the lowest level of “moderate to great” endorsement (47.8%), whereas the item assessing a greater appreciation for life received the highest endorsement (72.3%) (**Figure 1**).

### Qualitative Results

**Figure 2:**
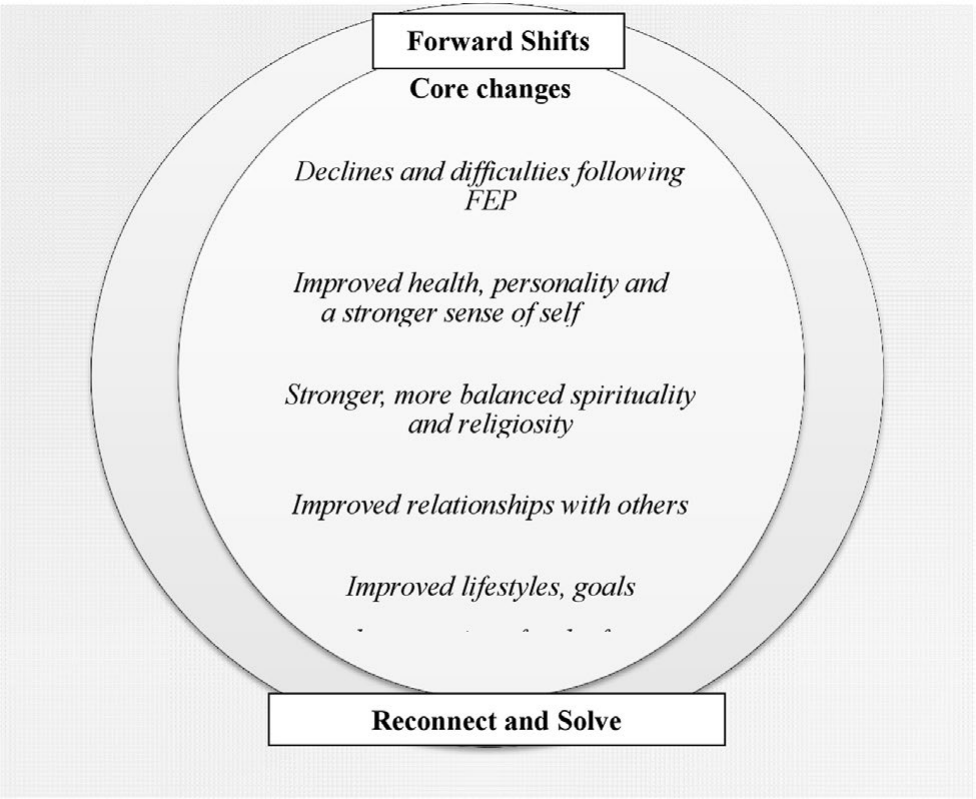
Changes experienced following the first episode of psychosis.

The thematic analysis produced three themes: life before and during the first episode of psychosis, declines and difficulties following psychosis, and experiences of positive change. Subthemes reflecting positive change included a) improved health, personality, and a stronger sense of self; b) stronger, more balanced religiosity and spirituality; c) improved relationships with others; and d) improved lifestyles, goals, and expectations for the future. Positive changes reflected fundamental forward shifts (i.e., new ways of being and functioning) from how participants were before they developed psychosis; efforts to reconnect with aspects of participants’ lives and selfhood that had once been important but were long forgotten; and attempts to solve or let go of past ways of being and living (**Figure 2**).

#### Theme 1: Life Before and During the First Episode of Psychosis

Many participants described experiencing overwhelming levels of stress, depression, numbness, and rumination, coupled with difficulties expressing their thoughts and feelings leading up to their psychosis. Many lived “wild” and often anxiety-provoking lifestyles. Some felt conflicted over their identities and personalities. Most described loving—but sometimes problematic—relationships with others. Others, however, felt cut off from relationships. Many were ambivalent about spirituality, whereas others were religious or intensely spiritual. Finally, many described experiencing difficulties with school or work.

Respondent: “I was really shut down. I was really afraid of … the opinions of others. I became very … withdrawn from society actually … Oh my God, just so different. Just really someone really shut down emotionally, unable to communicate, unable to express myself the way that I wanted to.”

Respondent: “I was wild. [Laughter]. I was wild…. I felt lost.”

#### Theme 2: Declines and Difficulties Following Psychosis

Following their first episode of psychosis, many experienced difficulties with emotions; acting on, structuring, or holding onto thoughts; communicating; taking care of themselves; knowing themselves as persons; relating to others; engaging in spirituality or religion; and working or being in school. Many also described struggling with the side effects of antipsychotic medication.

Respondent: “I lost hope and everything. I lost all my documents. Because it’s not, it’s not too long that I’ve been here in Canada, you know, and I have dreams, I have aspirations and it’s like I can’t fulfill these anymore…”

#### Theme 3: Experiences of Positive Change

##### Improved Health, Personality, and a Stronger Sense of Self

In addition to experiencing difficulties, participants described experiencing improved mental health and, to a lesser extent, physical health relative to how participants perceived themselves as having been before they developed psychosis. For instance, many described developing new perspectives, knowledge, understanding, and appreciation with respect to mental illness, mental health, mental health services, and the role they can play in recovery.

Respondent: “Yeah I learned my lesson. That’s a positive, learning my lesson. Stop, stay away from the drugs and stuff, after, like, so many years.”

Respondent: “I think I just wasn’t aware of it as much and … it was affected by psychosis because I never really had time … to have a lot of experiences with emotions because … this happened in the relatively early stages of my life, so … now … I can say that because of the tough situations I’ve been in, I’ve had an opportunity to be more aware of emotional intelligence.”

Participants spoke of experiencing improved psychological well-being following their first episode of psychosis. They described feeling better than before they developed psychosis and how their negative emotions gave way to positive emotions (e.g., happiness) and optimism, which had been rare before the onset of psychosis. Participants recounted experiencing improved ways of connecting with and feeling in control of their emotions. One said he had developed greater complexity of thought, whereas another said that she had begun thinking in pictures, which was helpful for writing, an activity she enjoyed. Some participants spoke of perceiving improvements in their physical appearance and health (e.g., by becoming “cleaner” and “more energetic”).

Respondent: “I’ve been less angry, less inpatient, and less anxious … and then a lot calmer and stuff. I think I can think things through. Before that, I was very impulsive. I acted on impulse and on stuff like that. I didn’t know what to do sometimes. I was just panicking.”

Respondent: “Well, I think I might have been…. All my life, I’ve never I’ve never felt as mentally stable as I have now.”

Participants described how they developed an improved capacity to promote or maintain their mental health and deal with challenges. Many felt that they had become more communicative, open, and willing to share their thoughts and emotions. Participants also learned to better identify and regulate their emotions, deal with stress and the unexpected, set aside time to spend with themselves, and let go of what they had realized did not matter. Many also stopped using substances and began taking responsibility for their own mental health.

Interviewer: And what about … how you deal with stress now, like since your psychosis?

Respondent: I cry. [Laughs] I just cry.

Interviewer: And before?

Respondent: How did I deal with it? Alcohol or a drug.

Respondent: “And for a problem, and let’s say a problem happens … like now, I don’t think ‘oh, I don’t have anything … there’s nothing I can do.’ Yes, there is something I can do. Right now, I’m thinking, ‘yes, there is something that I can do to improve my situation and to not be stuck to the feeling of being stuck…’”

Participants experienced positive changes with respect to their sense of self following psychosis. Commonly, participants perceived developing greater self-awareness and self-understanding. Some felt that they had gained self-worth and a greater awareness of their potential to transform into better people.

Respondent: “I realize that everything matters, like every word that comes out of my mouth, every thought that crosses my mind, like everything has an impact and everything makes a difference, and I didn’t have that awareness before.”

Respondent: “I felt even ashamed of myself…. And now I see nothing wrong. There’s nothing wrong with being blah, blah, blah, and that’s good.”

Often, participants mentioned developing a stronger sense of self. For some, this development was preceded by breaking off contact with abusive, toxic, or authoritative figures. Participants described how they had become more self-confident, self-assured, determined, and willing and able to take risks, pursue what they wanted from life, and face challenges.

Respondent: “And I think that who I am, and my sense of self right now is somebody who is a lot more confident, is a lot stronger, and values everything that makes my life possible a lot more than I used to. Because now I know what the alternative is, and the alternative is having … constant doubts as to whether or not I deserve to exist, based off the depression that came up…. And that means that … if I didn’t want to be here, I wouldn’t be here, so I should have that mentality going into everything that I do.”

Participants described refusing to be mistreated, committing to speak their mind even if it meant making others feel uncomfortable, and being resolute or committed to their choices.

Respondent: “before I used to pray, and I used to do all that stuff, and I thought, like, but you know it’s my life. That’s a big, big thing that I realized from psychosis—it’s my life.”

Several participants described how, following their psychosis, they became more self-accepting and “authentic” by becoming closer to their core, inner self, including their “old self.” They also described how they became more honest and truthful to their thoughts, feelings, and values.

Relatedly, one participant described becoming more grounded, which involved being connected to the world of everyday experience and emotion. Others described becoming more responsible, mature, and no longer acting “stupid.” Finally, some developed a more refined and kinder personality or identity, as well as improved values.

Respondent: “Well, it’s brought me like a lot closer to who I am and … yeah, it has changed my personality in the way that I feel like…. a much more open and receptive person where I was, before I was someone who was really just shut down and self-centered.”

Respondent: “I’m more like grounded and in like, practical lived experience mode, at the moment anyway, and I like that a lot better because I think I was like, too much in my mind and too much in my head before and I wasn’t connected to like my heart and I wasn’t connected to the ground, and now it’s like it all comes from the ground, from the bottom up, and it feels a lot better that way.”

##### Stronger, More Balanced Religiosity and Spirituality

Participants experienced several important changes in their spiritual and religious lives following the onset of psychosis. These included realizations that spirituality was real, yet ought to be connected to sound knowledge, verified wisdom, or common sense. Some developed new knowledge or understanding about spiritual, existential, and religious issues. Such knowledge included realizing the omnipotence of God and the omnipresence of spirituality.

Respondent: “Yeah and I also learned about, like God, where God is … It’s like you get a glass of water and you put some sand inside. We are the sand and God is, like the water … So … that is what I found out about where God is. So, he’s living amongst us and not somewhere in the sky.”

Respondent: “Just realizing that spirituality does exist and … there’s certain ways to go around it that have to, that need to make sense with everything else, you know. If it doesn’t line up with … sound knowledge of what we already know … if there’s no common thread of common sense … or some type of … verified wisdom, then it’s … most likely not relevant at that point.”

In addition, some participants developed new spiritual beliefs, such as in God. Relatedly, one participant described hearing the voice of God, which gave him comfort and a sense of security. It may be argued that this participant was finding solace in a delusional system and that, therefore, this may not constitute a true positive change. However, consistent with the constructivist paradigm, we acknowledged the possibility of multiple truths and chose to qualify these, as had the participant himself, as positive changes.

Interviewer: And what about religion or spirituality? Has your connection with religion or spirituality changed through psychosis?

Respondent: Uh, well, I believe in God now.

Interviewer: Before, you didn’t believe in God?

Respondent: I didn’t believe in anything.

Respondent: “Well, the voice that I hear … I can’t differentiate whether it’s a male or a female because it’s like it’s coming from the heart, which I believe it’s the voice of God. Like, each morning I get up, it tells me, like how are you and this and that and when I try to give up on my faith it will tell me ‘no, like there is something in store for you.’ Like, to keep on pressing, yeah.”

Participants also described how they engaged in new forms of participation in institutionalized religion or spirituality. Specifically, some described going to church more often and developing new connections with spiritual communities. Others described how they broke away from an organized religion that had been forced onto them by authority figures. Some also described engaging in new spiritual or religious rituals, including prayer and meditation.

Respondent: “I go every Sunday … to the church and I feel more closer to … to God and … I participate more in the [religious] community than before.”

Respondent: “Yes. Before I was with … He was a very religious person and very, how do you call, like, conventional religion … and … that wasn’t me at all … So, I broke out of that for sure and I just kind of … appreciate the unknown and believe that kind of, for now, the rest is kind of none of my business and I’m just here to live as fully as I can.”

Participants also described experiencing increased levels of guidance from religious or spiritual elements or being more willing to receive such guidance. For instance, one participant described a new willingness to relinquish control of the unpredictable to the will of God, and another spoke of following the guidelines of Christian moral conduct more closely. Similarly, one participant described how his spirit contained new knowledge that could offer him guidance in life. For another, such knowledge included information on the dangers of substance use.

Respondent: My spirit’s changed … I know what to do now instead of not knowing what to do … I know, like, not to go and do, like, 25 pills in one night anymore … Back in the day, I did it like I didn’t care. But now, I know … okay … I take X amount or take zero and I take zero…

Interviewer: And what kind of stuff do you feel that God has control over?

Respondent: I don’t know what is going to happen to me tomorrow. Maybe I’m going to have another episode … I don’t know that kind of stuff … I don’t know, I can’t control. I can do my best, but if it has to happen, it’s going to happen. And I have to be quiet and I have to … understand and accept that it’s happening and do my best.

Notably, some spoke of having developed more grounded and less zealous approaches to spirituality or religion that, they said, felt more congruent with being wise and maintaining a mental well-being.

Interviewer: Do you still tap into like religious or spiritual resources to deal with things?

Respondent: “With a different approach, I think with the correct approach, which is a more intelligent approach. Well, one that isn’t psychotic. Just realizing that spirituality does exist and there’s certain ways to go around it that have to, that need to make sense with everything else.”

##### Improved Relationships With Others

Many experienced improved relationships with others and with society following their psychosis. This included improved ways of thinking about and relating to others. For instance, participants spoke of realizing the value of others, the importance of relationships, and that they were loved and cared for by their families. Many developed improved relationships and made better friends. They felt that they became more considerate of other people’s needs; more receptive to the love and guidance of family; and more honest, easygoing, and comfortable around others. They also developed an improved capacity to deal with perceived negative aspects of other people; become kinder, more respectful and pleasant with others; as well as more humble, and less judgmental and pushy.

Respondent: “When I say we have better relationships, it’s like these prior months. It’s been like two, three months I feel like we have a better relationship because we’re seeing each other way more often … She always … been good to me. And I think it’s going to be even better in the future.”

Respondent: “Yeah…. I used to get mad too, with my mother a lot. I don’t anymore. I used to talk to her in a very rude way. I don’t anymore.”

Often, participants described improving the size and scope of their social networks and increasing the frequency of contact with loved ones. This entailed being with friends and family more often than before their psychosis, developing new friendships, and, in one case, gaining full custody of a child.

In contrast, participants spoke of relinquishing the need to fix other people’s problems, ceasing to worry too much about other people’s well-being, and learning to let loved ones learn from their own mistakes. Relatedly, many described letting go of unhealthy or superficial relationships such as with people whom they perceived as having exerted undue power and control over them.

Respondent: “At first it was difficult for me to … meet all my friends after my psychosis … because I had been in soin intensif [intensive care]…. They didn’t know all that. But after, I … just realize … and know who was my friends and what was the people that I wanted to tell them what happened … now I have the friends that are more close … I choose with who I want to be.”

Respondent: “I think it’s just how I saw the meltdown happen and the psychosis happen and the fact that I was in the hospital for a month, it just taught me that I can’t always think for others. I need to think about myself first.”

Finally, some also described no longer feeling bound by the expectations of other people or by the rules of society. However, one participant mentioned now recognizing the politics of social situations.

Respondent: “Like I needed to get a master’s degree, I needed to be in the biggest company, to be working for the biggest company in the world and I needed to that, just to be—just to feel successful and to—to be successful to other people. I don’t care about that anymore.”

Respondent: “I wouldn’t care, I would just go about my business and treat everybody normally and fairly, but I wouldn’t be sensitive to the status quo of the hierarchy, politics of the place. I wouldn’t be sensitive to that at all, but now I’m a lot more sensitive to that, I’ve realized it’s quite important in life to operate accordingly because it does affect a lot of things.”

##### Improved Lifestyles, Goals, and Expectations for the Future

Many reported improvements in their lives, having new goals, and new perspectives and understanding about life following their first episode of psychosis. Some developed a more positive perspective on life and a greater appreciation of life. Others realized that they wanted to change life directions and developed hope for the future, coupled with greater knowledge on how to achieve new goals.

Respondent: “I’m happier, in general…. Just appreciating life more in general because before that I was like no, I just want to die and stuff like that.”

Often, participants developed new activities, passions, pursuits, and goals. These included pleasurable activities such as going to restaurants more often, new occupational possibilities and intellectual pursuits, as well as constructive and prosocial activities aimed at strengthening community supports for others with mental health problems. One participant described integrating “fantasy type ideas about money” that developed during his psychosis into valuable goal-directed behavior.

Respondent: “Now I go much more to the cinema and we go out with my husband to restaurants and that kind of things.”

Many now engaged in preexisting activities with renewed passion and determination. They often described how their approach to art became more emotionally intelligent, broader, more thoughtful, and, for one participant, “deeper and darker.” One participant described how he had acquired new legitimacy as a social activist by drawing on the racism that he had experienced in the “magical realm” that was part of his “delusional” system. Some described how their tastes and interests—such as types of music—also changed to more closely reflect their values.

Interviewer: And has your … music changed in any way through experiencing psychosis compared to before the psychosis?

Respondent: I think it’s become darker and deeper.

Interviewer: In what sense deeper?

Respondent: Deeper as in it’s more … it reaches in further in terms of—I’ve had a lot of time to think about things, so it’s sort of more—the ideas are more sought after, they’re more explored than they previously were.

Respondent: I am very active in feminist scenes and … I don’t think that I could … understand the experience of other survivors unless I was one myself. And a vast majority of my abuse comes from this other world … and I feel like without those experiences, without … that kind of … rough, hardening … I would be very oblivious, or I would only have a textbook, theoretical perspective on what a lot of women go through. And … that means that I’d be speaking from another place of privilege.

Participants also felt happier with life. They felt more present and able to go with the flow of life and that they were constantly progressing and challenging their limits. Many participants learned to let go of aspects of their lives that they perceived as stressful, unimportant, or linked to arbitrary social expectations.

Respondent: “I’m on my way to always greater and better things. I just feel like in my life I’m just—I’m progressing so much, so fast. And every experience that I have just brings me more fulfillment and more enjoyment and more passion in my life and everything just keeps getting better and better.”

Respondent: “…and I don’t like drinking as much. And I went out last night, and I had a glass of wine, but that was it, you know? And I took sips off of my friend’s rum and cokes, but that was it. Like I don’t—I don’t like to drink to get drunk anymore, you know?”

Finally, participants described having new goals that they were working toward with greater focus. These ranged from occupational, educational, or financial goals to engaging in different forms of activism, for example, telling their story of mental illness to larger audiences.

Respondent: “My plan is to give $200 per pay, so $400 per month, to my student loan, to the government. I’ll be doing the same—200 per pay also—in a banking, in a savings account. And by the time I finish paying my student loan, I’ll have the same amount [in savings].”

Respondent: “…and from the first time I got to the hospital it was like I wanted to tell my story and to get my story out there, and finding different ways to do that and different ways to express myself…”

## Discussion

This study investigated aspects of positive change following a first episode of psychosis using mixed methods. This is the first investigation of positive change among persons receiving care at an early intervention service for psychosis using mixed methods. While acknowledging the significance of suffering, our study is important because it provides evidence that there may be more to the aftermath of FEP than suffering. Sharing this insight with young people experiencing a first episode of psychosis may provide them hope and a way out of their suffering. It is likely that the recovery-oriented nature of services provided by early intervention programs may play a role in promoting positive change. However, the extent to which clinicians at early intervention services are aware that such change is possible is unclear. Making clinicians aware that positive change is possible following FEP may help them validate and encourage experiences of growth among service users.

The quantitative results are consistent with other reports showing that positive change is possible following a range of adverse events and experiences (37–39). Studies of posttraumatic growth following other adversities have revealed that most people experienced positive change of a similar magnitude as in this study (40, 41). However, ours is the only study to report descriptive statistics on domains and items of the Posttraumatic Growth Inventory among persons having experienced an FEP. Notably, our qualitative findings are consistent with those observed in our review of other studies that, in studying recovery, found positive changes following FEP (4, 42). In explicitly focusing on positive change, our study therefore makes a substantial novel contribution. Our qualitative findings are also consistent with reports of positive change among persons who have experienced multiple episodes of psychosis (43).

We identified convergence and divergence between qualitative and quantitative findings. The qualitative findings revealed that positive changes are composed of new shifts in functioning, attempts to solve past mistakes, or attempts by participants to reconnect with forgotten aspects of themselves or their lives. We are unaware of any model or study of positive change that has described such manifestations of positive change following adversity. The quantitative results did not capture these nuances in positive change. Moreover, the qualitative findings captured what participants’ lives were like before and during the first episode of psychosis and the declines and difficulties that they experienced with the onset of psychosis. These findings were not captured in the quantitative results. An alternative version of the Posttraumatic Growth Inventory is available that measures posttraumatic growth and depreciation (44). Although this measure was not used in this study, several studies have confirmed that individuals experience both gains and declines following other types of adversity (45–47).

Spiritual change was an important and frequently reported area of positive change in participants’ narratives, which is consistent with other studies in FEP (4) and other adversities (48). Yet, spiritual change received the lowest endorsement of any domain on the Posttraumatic Growth Inventory, which too is consistent with studies focused on other adversities (49–51). This divergence suggests that the Posttraumatic Growth Inventory may not be sensitive to spiritual changes. Our qualitative interviews also captured an important aspect of spiritual change that is not measured by the Posttraumatic Growth Inventory—that of adopting a more balanced, less zealous approach to spirituality. This type of spiritual change has not been linked to spiritual growth following adversities (6–8, 52) and may reflect the extreme spiritual experiences that participants had during psychotic episodes or prodromal periods.

The items of the Posttraumatic Growth Inventory most endorsed by participants measured a greater appreciation for life following psychosis. This is consistent with studies in the context of other adversities (53, 54). During the interviews, however, participants seldom spoke of becoming more appreciative of their lives. Participants did, however, endorse changes in the qualitative component that may suggest a greater appreciation for life, even if not explicitly framed as such.

The qualitative findings identified improved health—in particular, mental health—as an important positive change, which is consistent with our systematic review (4). In contrast, the Posttraumatic Growth Inventory does not contain items that capture this type of change. This finding may reflect the context of the study. Participants may have described developing improved mental health because they were receiving care for psychosis at an early intervention service. This is consistent with other studies reporting improvements in domains of physical health following physical illnesses (45, 55).

The quantitative component revealed a moderate level of endorsement on items measuring the development of a stronger sense of self following FEP. Relatedly, the qualitative component captured changes to participants’ personalities, identities, traits, and levels of self-awareness. These individual-level areas of growth have been described in other studies that have focused on recovery in FEP (4) and are a common aspect of positive change across many types of adversity (56). Such changes may reflect a core reaction following adversity (6–8).

Similarly, the quantitative findings captured how relationships had improved following FEP, whereas the qualitative findings revealed how participants had learned to manage their relationships better and how the size and scope of their relationships were enhanced. Such changes are consistent with the model of posttraumatic growth (7). These changes have been described in studies of recovery in FEP (4) and may reflect participants’ perceptions that unhealthy relationships contributed to their psychosis (57).

Participants reported changed lifestyles and new goals and expectations following FEP, which is consistent with our review of positive change following FEP (4) and in studies of positive change following other adversities (57, 58). These findings may reflect participants’ realizations that the way they lived their lives earlier was not commensurate with their values or that some aspects of their lifestyles or society precipitated their psychosis.

Finally, some participants considered their new, ongoing psychotic experiences as useful additions to their lives in the qualitative component. This finding suggests that psychotic experiences, while often distressing, can be valuable and growth-promoting experiences in certain contexts. This finding is consistent with one qualitative study reporting posttraumatic growth among persons who had experienced multiple episodes of psychosis (43).

Given that most participants were emerging adults and that emerging adulthood is a developmental phase characterized by great volatility and experimentation with identity, work, views about the world (59), and so on, the positive changes that participants reported may have interacted with, or built upon, positive changes resulting from normative developmental processes.

### Implications

Our findings provide evidence that young people may change for the better following a first episode of psychosis. This insight can contribute to paradigm shifts that emphasize hope and possibility following experiences of mental illness. It can also help clinicians and early intervention services to implement interventions that foster growth, thereby more closely aligning services with the tenets of the recovery movement. Finally, results indicate the importance of experiencing high-quality treatment for mental illnesses.

### Strengths and Limitations

Our study used methods that were used with a high level of methodological rigor. Our use of mixed methods enriched our findings in ways that would not have been possible through a reliance on one method alone. Our study also included a well-characterized sample of service users.

However, we relied on a convenience sample of participants and on cross-sectional data collected at multiple time points over the course of participants’ follow-up in the quantitative component and relied on the treatment team to identify participants for the qualitative component.

### Future Directions

To establish the reliability and validity of quantitative assessments of positive change following FEP, future studies should investigate positive changes prospectively, conduct multiple assessments of positive change measured at specific time points following onset, and determine if positive change is related to improved mental health, well-being, and functioning. Future qualitative studies should investigate positive change following FEP in different treatment settings (e.g., peer support groups).

## Data Availability

The datasets for this study will not be made publicly available because we never obtained ethical approval to share people’s data.

## Ethics Statement

The study was approved by McGill University’s Research Ethics Board. All participants were explained the study and read and signed an informed consent form. They received copies of the consent forms.

## Author Contributions

GJ wrote the first draft of this manuscript, contributed to the design of the study, recruited participants, and analyzed the data. AM and SI helped draft the manuscript, contributed to the design of the study, and oversaw execution of the study.

## Conflict of Interest Statement

GJ is funded by CIHR and FRQS postdoctoral grants and has received funding from the McGill University Department of Psychiatry for his doctoral research. He has also received funding from the Franke Program in Science and the Humanities. AM is funded through the Canada Research Chair Program and has received funding from CIHR, FRQS, the National Institutes of Health (NIH), and Grand Challenges Canada. Unrelated to the present study, he has received research funding from BMS and Lundbeck as well as honoraria related to CME lectures, research consultation, and advisory board participation from Otsuka and Lundbeck. None of these pose any conflict of interest in relation to the present manuscript. SI has received funds from CIHR. She is funded by a CIHR New Investigator Salary award and previously received a salary award from FRQS.
